# Generalized trust as a foundation for online trust: findings from Austria, Greece, Poland, the Philippines, and South Africa

**DOI:** 10.3389/fsoc.2025.1504812

**Published:** 2025-04-07

**Authors:** Markus Hadler, Boštjan Vrečar, Rebecca Schaffer

**Affiliations:** Department of Sociology, University of Graz, Graz, Austria

**Keywords:** generalized trust, online trust, institutional trust, internet users, ISSP, digital societies

## Abstract

In this paper, we examine the relationship between generalized trust and online trust to assess whether the latter is a distinct phenomenon or an extension of the former. For this purpose, we provide an overview of different approaches developed to explain trust and discuss their applicability to online trust. Our analysis is based on a nationally representative sample of Austrians aged 16 or older, collected as part of the latest International Social Survey Programme (ISSP) survey on “Digital Societies,” as well as pretest data for this survey from Austria, Greece, Poland, the Philippines, and South Africa. Regression models considering indicators associated with a wide range of different approaches show that generalized trust is the strongest predictor of online trust. Hence, our findings suggest that online trust is not an independent concept but an extension of generalized trust, supporting the initial notion of generalized trust as a concept that goes beyond personal relationships, now also into the digital world.

## 1 Introduction

The concept of trust, including specific types such as social, generalized, and political trust, has gained significant prominence in the social sciences over the past few decades (Barbalet, 2019; Misztal, 2020; Vrečar, 2025). This growing interest coincides with the rapid rise of the Internet and online communication since the 1990s. Given that, many foundational studies on trust were conducted before the internet era (Rosenberg, 1956; Deutsch, 1958; Lewis and Weigert, 1985; Hardin, 1993; Yamagishi and Yamagishi, 1994; Earle and Cvetkovich, 1995; Fukuyama, 1995), questions arose, such as whether the internet positively or negatively affects trust (Uslaner, 2004) and how trust can be established in online environments (Hardin, 2004).

The present research also addresses the relationship between online and offline trust, but asks a more fundamental question of whether online trust is a distinct phenomenon or a mere extension of offline trust. This question adds notably to the existing literature on digital environments, which focuses often on specific aspects of online trust, such as consumer behavior, reputation systems, and internet transactions (Yoon, 2002; Wang and Emurian, 2005; Mutz, 2005). Additionally, studies frequently investigate specific online interactions, including reputation in internet markets (Khopkar and Resnick, 2009), gaming (Lundmark, 2015; Chen et al., 2016), experiences of discrimination (Näsi et al., 2017), negative impacts of online interactions (Näsi et al., 2015; Pavić and Šundalić, 2016; Hergueux et al., 2021), online trust’s relation to social capital (Zhou et al., 2022), and other topics.

Research on the foundations of online trust is less common. Furthermore, existing literature shows that this research draws on offline trust concepts: For instance, Beldad et al. (2010) link online trust in both commercial and non-commercial contexts to classic offline theories, such as psychological approaches and attitudes. Similarly, Taddeo (2010) applies a rational choice framework, while Antonijević and Gurak (2019) start from Luhmann’s theories. Melanie Green (2007) also adopts a psychological perspective when observing that respondents report significantly lower trust in people on the Internet compared to people in general. We consider generalized trust as an additional source of online trust and build in this regard on insights from research on offline trust that considered the relations between different types of trust, such as Freitag and Traunmüller (2009), Newton and Zmerli (2011), Glanville and Shi (2020), Zheng et al. (2023), and Fairbrother et al. (2024).

Given that the above-mentioned literature on the sources of online trust suggests that online trust shares a similar foundation with offline trust, we first present key theoretical assumptions and their potential extensions to online trust, based on prior reviews by Delhey and Newton (2003, 2005), Nannestad (2008), Delhey et al. (2011), Newton et al. (2018), Schilke et al. (2021), and Vrečar (2025). The different approaches depicted in Table 1 inform the selection of items for our subsequent analysis of the sources of trust. Table 1, therefore, also includes several items, which will be discussed in greater detail in the methods section.

**Table 1:** Theoretical approaches to trust and related independent variables.

Theoretical approaches	Main arguments	Variables and Variable name (see Methods section)
Personality perspective (Erikson, 1950; Rosenberg, 1956; Allport, 1979; Uslaner, 2002, 2018)	Trust is a mostly persistent personality trait learned in childhood, later experience (e.g., traumatic, collective) can potentially influence it.	Personal optimism (A2), general optimism (for the country) (A3), gen. Life-satisfaction (A1), a sense of control over life (A6/item 6,7; F16/item 5), general subj. Health (F24), chronic health issues (F 25), general happiness (F1), socio-demographics (gender D1, age D2_1, education level D3_1, unemployed D4 answer 4, personal income D12, net household income D13, ethnicity_ birthplace D31, ethn: birthplace father D32, ethn; birthplace mother D33, residential area_size D34, religious affiliation D26)
Civic culture and values perspective (Almond and Verba, 1963; Putnam, 1993, 2000; Brehm and Rahn, 1997; Fukuyama, 1995; Rahn and Transue, 1998; Paxton, 2007; Paxton and Ressler, 2018)	Trust is connected with moral norms, extensive (in)formal networks of associations, and (inclusive) participative culture and values differences among groups or generations—e.g. in egalitarianism, (post-)materialism, or religious heritage such as Protestantism.	Online bridging vs. online bonding behavior (Q 15/item 1,2), online/offline sociopolitical participation (Q23/all 5 items), egalitarian attitudes (inheritance tax) (A9), religious affiliation (D26/dummy for answers 2,3,4), solidarity attitudes (F5), political left–right self-positioning (Q24), last parliament election voting (D29), plus socio-demographics (see above)
Rational choice perspective (Gambetta, 1988; Coleman, 1990; Hardin, 1993, 2002; Robbins, 2016; Cook and Santana, 2018)	Trust is based on the reasoning and experience of individuals, their encapsulation of mutual interests, and probabilistic evaluations of others´ trustworthiness and positive outcomes.	Experience of online fraud (Q18/dummy for answers 1,2,3), experience of online mobbing or discrimination (Q19/dummy for answers 1,2,3)
Societal status/ class perspective (Patterson, 1999; Wilkinson, 2005; Rothstein and Uslaner, 2005; Bauer and Freitag, 2018; Dinesen and Sønderskov, 2018)	Trust reflects structural characteristics of societies and inequalities connected to status, class, race/ethnicity, inherent divisions, polarizations and conflicts. Higher-status “winners” and “haves” tend and can afford to trust more than “losers,” “have-nots” or minority groups with a history of discrimination.	Migration_ethnicity (D31, D32, D33), income_class (D12, D13, D14), job status (D4), education status (D3_1, D3_2), plus other sociodemographics (see above)
Institutional perspective (Levi, 1998; Rothstein and Stolle, 2001, 2008; Möllering, 2005)	Trust, above all generalized social trust, is associated with the quality of government and “good” institutions through their (perceived) corruption, fairness, impartiality, and trust in state institutions, esp. law enforcement.	Political trust in law-and-order institutions (judiciary) (Q 26/1), trust in representative institutions (parliament) (Q26/2), institutional trust in media (Q21/item 1), institutional trust_health system (F2)
Social constructivist perspective (Larsen, 2013; Frederiksen, 2019)	Trust is associated with one’s perception of society, its structure, conflicts, inequalities, of oneself, other individuals and groups.	Sociopolitical (self-)perception_social conflict (A6/item 5), self-positioning_belonging to a discriminated social group (A4), class self-positioning in the society_above-below (A7, A8)

Sources: Delhey and Newton (2003), Nannestad (2008), Newton et al. (2018), and Schilke et al. (2021).

The theories depicted in Table 1 can be divided into approaches focused (a) predominantly on individuals and their traits, perceptions, and evaluations and (b) approaches focused on societies and cultures and their institutional contexts and structural elements. The first individual-level approach is the *personality perspective*, which posits that trust is a stable trait, largely established in childhood and influenced by early life experiences (Erikson, 1950; Uslaner, 2002). It suggests that trust, whether in online or offline contexts, is associated with a positive outlook on life, including factors such as personal optimism, life satisfaction, and a sense of control. Trust, in this view, remains consistent across different domains, with online experiences having little impact on an individual’s overall level of trust (Uslaner, 2004). The *rational choice perspective* is another individual-focused approach. It considers trust as a product of cognitive evaluations and experiences. Individuals calculate the likelihood of others being trustworthy based on their past interactions. Positive experiences, particularly in repeated interactions, tend to increase trust, while negative experiences reduce it (Coleman, 1990; Gambetta, 1988). Trust in this context is seen as a rational decision, shaped by the perceived trustworthiness of others and the expected outcomes of trusting relationships (Hardin, 2002). Finally, the *social constructivist perspective* also emphasizes the role of individual perceptions in shaping trust. Trust is seen as influenced by how people perceive societal structures, conflicts, and inequalities (Larsen, 2013; Frederiksen, 2019). Individuals who view their society as predominantly egalitarian and middle-class, not eroded with cleavages and divisions, are more likely to exhibit high levels of generalized social trust, reflecting their belief in the fairness and stability of their social environment.

Contextual elements are emphasized in the *civic culture and values* perspective, which links trust to broader cultural processes and civic values. Trust is seen as emerging from democratic traditions, civic engagement, and social capital, which includes networks and norms of reciprocity (Putnam, 1993, 2000; Fukuyama, 1995). This approach suggests that individuals with strong bridging social capital and participative behaviors, both online and offline, are more likely to exhibit high levels of trust. Additionally, trust correlates with egalitarian attitudes and religious backgrounds, particularly Protestantism (Fukuyama, 1995: 283–6; Delhey and Newton, 2005: 318–320). The *societal status/ class perspective* interprets trust as reflecting the structural characteristics of society, including class, status, and ethnic divisions. Trust is more prevalent among those with higher socioeconomic status, who are more likely to experience stability and security (Patterson, 1999; Wilkinson, 2005; Rothstein and Uslaner, 2005). In the online world, this perspective would suggest that individuals who are better educated, wealthier, and more digitally literate are more inclined to trust others, as they are more familiar with and confident in navigating digital environments. Finally, from an *institutional perspective*, trust is closely tied to the quality of government and institutional fairness. Trust in others, including in online contexts, often stems from trust in political institutions, particularly those perceived as fair and impartial. This approach posits that individuals who see democratic state institutions as mostly fair and therefore trust them, especially those implementing policy, such as courts and their public officials, are more likely to extend that trust to fellow citizens and strangers, both online and offline (Rothstein and Stolle, 2008).

As mentioned earlier, this research examines the sources of online trust and its relationship with offline trust. More specifically, we focus on generalized trust as the survey we use in our analysis asked about trust in general and not about trust in particular individuals or groups. The literature allows for two opposing perspectives on this relationship between online and offline trust: On one hand, the core idea of generalized trust—the belief that “most people can be trusted” and that trust extends beyond direct, personal interactions (Stolle, 2002; Uslaner, 2002)—suggests that it also extends to online contexts, making online trust a subset of generalized trust. On the other hand, studies on distinctions between generalized and particularized offline trust (Freitag and Traunmüller, 2009), social and political trust (Newton and Zmerli, 2011), and the transition from particular to generalized trust (Glanville and Shi, 2020; Zheng et al., 2023) were also able to demonstrate that trust can take on distinct types. Applying these findings to the relationship between online and offline trust opens the alternative possibility that online trust may represent a separate and unique type of trust.

## 2 Methods

Our analyses are based on questions from the new International Social Survey Programme^1^ survey on “Digital Societies,” the ISSP survey on “Health and Health Systems,” and a few country-specific questions that were fielded in a single survey in Austria. The survey is representative of the Austrian population aged 16 and older and was conducted in early 2024. The sample was drawn randomly from the national registry and 3,800 people were invited via postal mail to complete the survey either online or, after two reminders, in a printed mail-in format. The final sample size was 1,546 respondents, which corresponds to a response rate of 40.7%. Our analysis is limited to internet users, as one of the dependent variables is about online trust and was only captured from internet users. Our final sample size is thus 1,434 cases. The data (Hadler et al., 2024) is available at the Austrian Social Science Archive (AUSSDA).

Furthermore, we used the pre-test data from the development of the ISSP Digital Society survey from 2022, which includes samples from Austria, Greece, Poland, the Philippines, and South Africa. These samples did not include all questions of the final survey and followed less strict data collection rules. Therefore, only use them to tentatively check whether our findings on the relation between generalized trust and online trust can also be observed in other countries. Here, the total sample size is 4,055. This second dataset is also available at AUSSDA for replication purposes (Andreadis et al., 2024).

Our dependent variables on trust are measured in the following way: (a) “Generally speaking, would you say that most people can be trusted or that you cannot be too careful in dealing with people? Please tick ONE box to show what you think, where 0 means you cannot be too careful, and 10 means most people can be trusted.” This question on generalized trust uses the wording that was introduced in the US General Social Survey in 1972 and has been used ever since widely since then in various surveys including the ISSP, despite debate about its measurement properties (see for example Reeskens and Hooghe, 2008).

The ISSP group developed a similar question for online contacts for its “Digital Societies” survey: (a) “On a scale of 0 to 10, how much do you trust people you are communicating with on the Internet but have never met in person? 0 means you do not trust them at all, and 10 means you trust them completely” with the same answer categories as for the generalized trust. The wording “never met in person” was added after the pre-test to emphasize the online-only aspect and to exclude communication with family, close friends, etc. Table 2 shows the descriptive statistics for these two variables and for all independent variables. The independent variables were selected based on all items in the survey that are related to the different approaches displayed in Table 1.

**Table 2:** Descriptive statistics (internet users only).

Variable name and wording (translated from German)	Statistics (min/max/mean or median/st.dev. or %)
Q25: In general, what do you think: can you trust people or can you not be careful enough when dealing with people?	Min.: 0 you cannot be too careful; Max.: 10 most people can be trusted; Mean: 4.54; Std.dev.: 2.564
Q14: On a scale of 0 to 10, how much do you trust people you communicate with over the Internet but have never met in person?	Min.: 0 no trust at all; Max.: 10 complete trust; Mean: 2.3; Std.dev.: 2.233
Personality perspective	
A2: Do you think your personal circumstances will change in the next few years?	Min.: 1 deteriorate significantly; Max.: 5—improve significantly; Med.: 3
A3: Do you think living conditions in Austria will change in the next few years?	Min.: 1 deteriorate significantly; Max.: 5—improve significantly; Med.: 2
A1: How satisfied are you with your life overall at the moment?	Min.: 1 completely satisfied; Max.: 7 -completely dissatisfied; Med.: 3
A6_6: To what extent do the following statements apply to you?—I believe that I am free to decide how I live my life.	Min.: 1 applies very much; Max.: 5 does not apply at all; Med.: 2
A6_7: To what extent do the following statements apply to you?—Life has become so complicated these days that I can hardly find my way around.	Min.: 1 applies very much; Max.: 5 does not apply at; Med.: 4
F16_5: Please think about the last 4 weeks. How often…—have you had the feeling that you could not cope with your problems?	Min.: 1 never; Max.: 5 very often; Med.: 2
F24: All in all, would you say your health is….	Min.: 1 excellent; Max.: 5 poor; Med.: 3
F25: Do you have a long-term illness, a persistent health problem or a disability?	1—yes 28.8%; 2—no 71.2%
F1: If you look at your life today: How happy or unhappy are you all in all?	Min.: 1 completely happy; Max.: 7 completely unhappy; Med.: 3
D1: Gender	1 male 49.4%; 2 female 50.6%
D2_1: When were you born? Please enter your year of birth.	Min.: 1931; Max.: 2007; Mean: 1976; Std.dev.: 17.998
D34: Where do you live?	Min.: 1 in a large city; Max.: 6 in a detached house or farm in the countryside; Med.: 4
Civic culture and values perspective	
Q15_1: Has your online contact with the following people increased, decreased or remained the same in the last 12 months?—With people who share your political views	Min.: 1 decreased significantly; Max.: 5 increased significantly; Med.: 3
Q15_2: Has your online contact with the following people increased, decreased or remained the same in the last 12 months?—With people who do not share your political views	Min.: 1 decreased significantly; Max.: 5 increased significantly; Med.: 3
Q23_1: Below you will find various forms of political and social activities that people can engage in. For each of these activities, please indicate whether you have done them in the only offline, only online or both offline and online in the last 12 months: Participated in a signature campaign	1 only offline 8.4%; 2 only online 18.8%; 3 both, offline and online 11.8%; 4 not at all, neither offline nor online 61%
Q23_2: […] Participated in a demonstration or political protest	1 only offline 6.7%; 2 only online 2.9%; 3 both, offline and online 2.7%; 4 not at all, neither offline nor online 87.7%
Q23_3: […] Contacted or attempted to contact politicians to express your opinion	1 only offline 3.5%; 2 only online 3.9%; 3 both, offline and online 2%; 4 not at all, neither offline nor online 90.6%
Q23_4: […] Organized or helped to organize a demonstration or political protest	1 only offline 0.9%; 2 only online 1.1%; 3 both, offline and online 1.5%; 4 not at all, neither offline nor online 96.6%
Q23_5: […] Belonging to or joining a group that campaigns for social issues or is committed to a cause	1 only offline 6.4%; 2 only online 5.4%; 3 both, offline and online 4.4%; 4 not at all, neither offline nor online 83.9%
A9: And what do you think about inheritance tax? Which of these would you consider good?	Min.: 1 all inheritances should be taxed; Max.: 6 inheritances should not be taxed; Med.: 5
D26: To which religious community do you belong?	1 christian community 62.6%; 2 evangelical religious community 3.3%; 3 non-Christian community 5.9%; 4 no religious community 28.2%
F5: To what extent would you be prepared to pay higher taxes in order to improve healthcare for everyone in Austria?	Min.: 1 definitely ready; Max.: 5 not ready at all; Med.: 4
Q24: In politics, people sometimes talk about ‘left’ and ‘right’. Where would you place yourself on a scale of 0 to 10, where 0 means ‘left’ and 10 means ‘right’?	Min.: 0 left; Max.: 10 right; Mean: 4.52; Std.dev.: 2.146
D29: Who did you vote for?	1 ÖVP (Conservative) 35.5%; 2 SPÖ (Social Democrats) 23.9%; 3 FPÖ (right wing) 12.4%; 4 GRÜNE 19.3% (Greens); 5 NEOS (liberal) 5.3%; 6 Other 3.6%
Rational choice perspective	
Q18: Some people receive emails or text messages from scammers from time to time, some of them also become victims of online fraud. Apart from just receiving such messages, have you or someone you know personally ever been the victim of online fraud, identity theft or hacking?	1 yes, myself 12.4%; 2 yes, someone I personally know 29.7%; 3 yes, both myself and someone I personally know 5.8%; 4 no 52%
Q19: Manche Menschen berichten über Erfahrungen mit Belästigungen im Internet. Haben Sie selbst oder jemand, den Sie persönlich kennen, jemals Belästigungen im Internet erlebt oder waren Opfer von Hassreden im Internet?	1 yes, myself 8.1%; 2 yes, someone I personally know 14.4%; 3 yes, both myself and someone I personally know 6.1%; 4 no 71.4%
Societal status-class perspective	
D31: Where were you born?	1 Central Europe 89%; 2 South-eastern Europe/Balkans 2.9%; 3 other countries 8%
D32: Where was your father born?	1 central Europe 83.3%; 2 South-eastern Europe/Balkans 6.3%; 3 other countries 10.3%
D33: Where was your mother born?	1 central Europe 82.8%; 2 South-eastern Europe/Balkans 5.9%; 3 other countries 11.3%
D12: What is your current personal monthly net income? (categorized)	Min.: 0 No personal income; Max.: 11 over 5.000 euro; Mean: 3.78; Std.dev.: 2.708
D13: Now please consider all income of all household members: earned income, pensions, social benefits (e.g. family allowance), regular private cash benefits, etc. Can you indicate how much income is available to your household NET per month BEFORE deducting any expenses such as rent, etc.?	Min.: 0—No household income; Max.: 11—over 5.000 euro; Mean: 6.46; Std.dev.: 3.676
D14: Please think about your total household income, i.e., from all sources of income and from all people who contribute to it. How difficult or easy is it currently for your household to make ends meet?	Min.: 1 very difficult; Max.: 5 very easy; Med.: 3
D4: Are you currently primarily…?	1 employed or working 58.4%; 2 not employed or job-seeking 41.6%
D3_1: What is your highest school qualification?	Min.: 1—No school diploma; Max.: 16 Other qualification after the Matura (e.g., university); Med.: 3
D3_2: How many years in total were you in school, university or other school-based training, without in-company training?	Min.: 1; Max.: 61; Mean: 12.07; Std.dev.: 3.761
Institutional perspective	
Q26_1: On a scale from 0 to 10, how much trust do you have in each of the following institutions?—In the Austrian parliament	Min.: 0 no trust at all; Max.: 10 complete trust; Mean: 4.16; Std.dev.: 2.702
Q26_2: On a scale from 0 to 10, how much trust do you have in each of the following institutions?—In the Austrian courts	Min.: 0 no trust at all; Max.: 10 complete trust; Mean: 5.9; Std.dev.: 2.750
Q21_1: In your opinion, how reliable is the news on political issues that you find in the following sources?—Social media (e.g. WhatsApp, Facebook, Tiktok, Instagram, YouTube, Telegram)	Min.: 1 very unreliable; Max.: 4 very reliable; Med.: 2
F2: How much confidence do you generally have in the healthcare system in Austria?	Min.: 1 complete trust; Max.: 5 no trust at all; Mean: 2.47; Std.dev.: 0.84
Social constructivist perspective	
A6_5: To what extent do the following statements apply to you?—There is a conflict in Austria between the ordinary people and the ruling elites.	Min.: 1 applies very much; Max.: 5 does not apply at all; Med.: 2
A4: What do you think, are you yourself one of the people or a group of people in our society who are …	Min.: 1 highly disadvantaged; Max.: 5 highly privileged; Med.: 3
A7: If you think about yourself, where would you place yourself on this scale of 1 to 10? 1 means “bottom” and 10 means “top.”	Min.: 1 below; Max.: 10 above; Mean: 5.86; Std.dev.: 1.714
A8: And when you think about the family you grew up in, where would they have been categorized back then?	Min.: 1 below; Max.: 10 above; Mean: 5.49; Std.dev.: 2.042

Source: Austrian sample of the ISSP Digital Societies survey and Austria-specific questions. See the Methods section for details.

Our analytical approach is as follows. Initially, we present descriptive results on the two trust variables and their correlations. This is followed by different regression analyses. Given the large number of indicators associated with the different theory approaches, we explored several reduction strategies. We considered factor analyses, which are suitable only for correlated variables and not applicable to socio-demographic variables and other sets of indicators associated with the different approaches. We also explored a stepwise regression, which adds variables based on their explanatory power. However, this method can introduce biases, such as variable omission or overfitting.

Ultimately, we adopted the following two-step approach. In the first step, we ran separate regressions with either generalized trust or online trust as the dependent variable and all independent variables associated with a single theoretical approach (e.g., personality, civic culture, etc.). We recorded which variables remained significant in each combination of trust and theory approach. In the second step, we ran regressions that included all variables identified as significant in these initial regressions. Additionally, we cross-validated the results using alternative approaches, such as regressions including all variables and the stepwise regression mentioned earlier. While the results show slight variations across methods, the main conclusion regarding the relationship between offline and online trust remained consistent.

Finally, before presenting the results of our analyses, we must place our study in a societal context for better interpretation. Austria is considered a medium-trust society (Delhey and Newton, 2005: 315). During the 1990s and early 2000s, social trust, as measured by the “most people can be trusted” question, was around 30 percent and above, according to the integrated World Values Survey Data. Since then, trust has steadily increased to over 40 percent, despite Austria’s rapidly growing ethnic diversity in the past two decades. Similar trends are observed in the ISSP data, where generalized social trust averages around 50 percent on a scale from 0 to 10, reflecting a general faith in fellow citizens (Hadler et al., 2020). Regardless, as pointed out above, we will use the international pre-test dataset to verify some of our national results and check if we see similar findings in other countries.

## 3 Results

**Figure 1:**
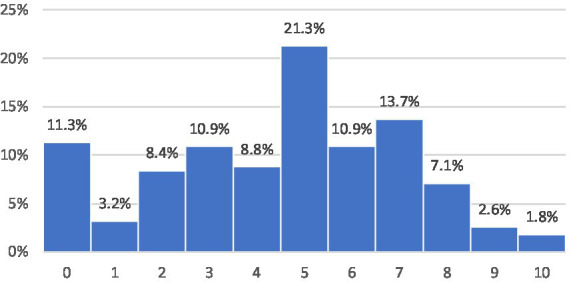
Generalized Trust (internet users only). Formulation of the question: “In general, what do you think: can you trust people or can you not be careful enough when dealing with people? Please check ONE box to show what you think; where 0 means you cannot be too careful and 10 means you can trust most people.” Source: Austrian sample of the ISSP Digital Societies survey and Austria-specific questions. See the Methods section for details.

**Figure 2:**
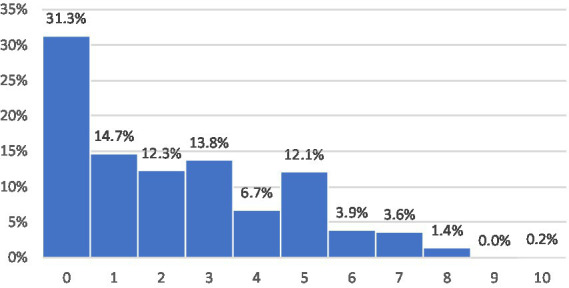
Online Trust (internet users only). Formulation of the question: “On a scale of 0 to 10, how much do you trust people you communicate with over the Internet but have never met in person? 0 means you have no trust at all, and 10 means you have complete trust.” Source: Austrian sample of the ISSP Digital Societies survey and Austria-specific questions. See the Methods section for details.

As described in the data section, we first present some basic results on offline and online trust. Figure 1 shows the level of generalized trust and Figure 2 the online trust in the Austrian data from 2024. Both figures are limited to internet users. The distributions show that the online trust is much lower with a large group of respondents indicating no trust at all. This rather low trust is also due to the question wording, which asks specifically for people who you usually do not communicate with. The pre-test data (Andreadis et al., 2024) did not include the term “stranger,” which resulted in a higher level of trust. Regardless of the magnitude, the overlap between offline and online trust is moderate with an overall Pearson correlation of 0.221 in the Austrian data and an overall correlation of 0.164 in the international dataset. The subsequent regression analyses will further explore this question and examine the underlying factors that shape trust.

Table 3 presents the results of the regression analyses. As described in the methods section, the included variables were selected based on their significance when considering only a single theory approach. Model 1, on generalized offline trust, shows that trust in institutions—more specifically in Austrian courts—has the strongest effect with a Beta of 0.198. The feeling of control over life’s problems, related to the personality perspective, is also significant with a Beta of 0.173. In addition, variables related to societal status and the social constructivist view are significant, i.e., residency and household income. Education fails to be significant at a 0.05 level. All other variables are not significant in this model, hence, generalized trust is mostly influenced by factors related to the institutional, personality, as well as to the social status and social constructivist view.

**Table 3:** Regression models on generalized and online trust (Beta values).

Variables	Model 1 General Trust	Model 2 Online Trust	Model 3 Online Trust
Gender –Female (RC = male)	x	−0.163**	−0.169**
Age (old—young)	x	0.140**	0.132*
Residence (city-country)	0.109*	x	−0.017
Education (low-high)	0.095^+^	x	0.003
Household net income (none—over 5.000 euro)	−0.102^+^	x	−0.005
Living on household net income (difficult—easy)	0.118*	x	−0.005
General offline trust (no trust—complete trust)	x	X	0.214**
Political trust in courts (no trust—complete trust)	0.198*	x	−0.052
Political trust in parliament (no trust -complete trust)	0.088	0.119**	0.100
Trust in social media regarding political issues (no trust -complete trust)	x	0.134**	0.129*
Affiliation to population group (disadvantaged—advantaged)	0.012	0.082*	0.062
Online contact with politically like-minded people (decreased -increased)	x	0.116**	0.110*
Complexity of life (applies—does not apply)	0.173*	x	−0.049
Difficulty managing problems (never—very often)	0.097^+^	0.080*	0.083
Victim of harassment/hate speech—Yes, myself (RC = no)	−0.033	0.098*	0.101*
R^2^	0.193	0.144	0.145

*N* = 1,434. Regression 1: DV = general offline trust; Regression 2: DV = online trust; Regression 3: DV = online trust, IV = general offline trust. Significance level: +*p* < 0.1, * *p* < 0.05 = significant; **p < 0.01 = highly significant; x = not included in the regression; beta values are shown. RC = Reference Category. Included but not significant in any model: Voting Preference, Trust in Web Sites, Health Condition, Happiness, Minority Status, Subjective Social Position, Subjective Social Position of Family of Orientation, Conflict Perception, Future Living Outlook, Victim of Fraud, Victim of Harassment (category someone else). Source: Austrian sample of the ISSP Digital Societies survey and Austria-specific questions. See the Methods section for details.

Model 2 presents the results for online trust. Online trust is also shaped by institutional trust, in this case, trust in the Austrian parliament (Beta = 0.119). Compared to offline social trust, online trust is also influenced by other online trust indicators such as trust in the accuracy of social media (Beta = 0.134). Furthermore, sociodemographic characteristics, i.e., being young (Beta = 0.140), gender (−0.163), and belonging to a disadvantaged social group (0.082), are significant, which supports approaches considering social class and constructivist views. Furthermore, political bonding online (civic perspective) is significant (Beta = 0.116), as is being a victim of online harassment (reflecting experiential rational choice view) with a Beta of 0.098. The rational choice indicator of being a victim of online harassment has surprisingly a positive effect. A possible explanation is that the cause-and-effect direction might be reversed and trusting individuals might get in contact with perpetrators more often. Finally, similar to general trust, the issue with managing problems has a positive effect. Surprisingly, its effect is opposed to its empowering impact on generalized offline trust in the initial model. However, it shows that online trust is also influenced by personality factors. In sum, this first analysis of online trust suggests that online trust is influenced by factors associated with all approaches shown in Table 1.

To explore the relationship between online trust and generalized trust, we added the latter as an additional independent variable to Model 2. The results are shown in Model 3. The impact of sociodemographic characteristics—trusting more when young and male—remains significant. The phenomenon of political bonding also retains its relevance for online trust (Beta = 0.110)—a finding that supports the proposition that on the Internet, we may also be confronted with a distinct and more particularized dimension of trust, as engaging online with politically like-individuals results in higher trust. Factors related to social class/status, social constructivist views, and the civic perspective also remain strong. Similarly, the rational choice-related indicator and online harassment remain significant. A larger shift happens regarding trust indicators not related to the online sphere. Model 3 shows that generalized trust becomes the strongest predictor for online trust (Beta = 0.214), whereas trust in the institution “parliament” is not significant anymore. This finding supports the view that online trust is strongly related to generalized trust despite the mixed results in our initial descriptive analyses.

To verify these findings of Model 3, we estimated a similar regression with the international dataset. We included all significant indicators of Model 3 except for the difficulty managing problems indicator, which was not included in the pretest data, and added the trust in institutions indicator “parliament” and fixed effects for each country. This verifying regression confirms that generalized trust (Beta = 0.115**) has a stronger effect than institutional trust (Beta Parliament = 0.101**; Beta Social Media = 0.108**). Generalized offline trust, hence, is central to online trust, which is also underscored in another additional analysis, a factor analysis, which shows that online and offline trust and trust in a parliament form a single factor with generalized trust having the strongest loading.

## 4 Discussion and conclusion

The present research note explores the sources of online trust with a focus on generalized trust. The initial literature review showed that scholars addressing the sources of online trust (Beldad et al., 2010; Taddeo, 2010; Bouchillon, 2014; Antonijević and Gurak, 2019) also build on classic theories developed in an offline setting. Therefore, we presented six theoretical perspectives that highlight different sources of trust. The approaches include the personality perspective, associated with scholars such as Erikson (1950), Rosenberg (1956), Allport (1979), and Uslaner (2002, 2018), the civic culture and values perspective, studied by Almond and Verba (1963), Putnam (1993, 2000), Brehm and Rahn (1997), Fukuyama (1995), Paxton (2007), and Paxton and Ressler (2018), the rational choice perspective, as addressed by Gambetta (1988), Coleman (1990), Hardin (1993, 2002), and Cook and Santana (2018), the societal status/class perspective, associated with Patterson (1999), Wilkinson (2005), Rothstein and Uslaner (2005), and Dinesen and Sønderskov (2018), the institutional perspective, as discussed by Levi (1998), along with Rothstein and Stolle (2001, 2008), and, lastly, the social constructivist perspective covered by Larsen (2013) and Frederiksen (2019).

As for the relationship between offline and online trust, we considered two possibilities. The basic premise of generalized trust—the belief that “most people can be trusted” and that trust extends beyond personal interactions (Stolle, 2002; Uslaner, 2002)—suggests that online trust is a subset of it. However, research on offline trust was also able to differentiate between generalized and particularized trust (Freitag and Traunmüller, 2009), social and political trust (Newton and Zmerli, 2011), and transitions from particular to generalized trust (Glanville and Shi, 2020; Zheng et al., 2023), which also allows for the alternative view that offline and online trust are distinct concepts.

Using survey data from Austria and four other countries, we identified questions and items that relate to these six theoretical perspectives mentioned above and tested their effect on offline and online trust. In the separate models of offline and online trust, the institutional perspective emerged as the most influential theoretical approach, which is consistent with some recent experimental and cross-country trust research (Martinangeli et al., 2024; Domański and Pokropek, 2021). However, when adding generalized offline trust to the regressions on online trust, its effects are stronger than those of the institutional trust indicators. Furthermore, an additional factor analysis showed that online trust and generalized trust load were on the same dimension, whereas the loading of generalized trust load was stronger. These findings show that generalized offline trust is central to online trust. In this sense, these findings rather point to online trust as an extension of generalized trust than to an independent concept and suggest that the belief that “most people can be trusted” and that trust extends beyond personal interactions (Stolle, 2002; Uslaner, 2002) also applies to the link between an offline and an online environment.

In conclusion, this research note offers some first insights into the relationship between online and offline trust using the forthcoming ISSP “Digital Societies” survey. Yet, we face some limitations. At this point, we have data only for a few countries. Further, the questions asked in the survey, and the match between the indicators and the theories were not always perfect. Third, we relied on cross-sectional data, which precludes us from examining causality—such as whether trust in online contacts is a cause, effect, or both, of online harassment (or its absence). Future research could address these limitations by analyzing a broader range of countries, once the ISSP releases the full dataset with up to 45 countries in 2026, and exploring alternative datasets or methods, such as combining survey data with external indicators aligned with the theoretical frameworks.

## Data Availability

Publicly available datasets were analyzed in this study. This data can be found here: Hadler et al. (2024) and Andreadis et al. (2024).

## Footnotes

^1^ www.issp.org
